# Dystrophic Epidermolysis Bullosa in Pregnancy: A Case Report of the Autosomal Dominant Subtype and Review of the Literature

**DOI:** 10.1155/2014/242046

**Published:** 2014-04-24

**Authors:** Nicole Colgrove, Rayan Elkattah, Howard Herrell

**Affiliations:** Department of Obstetrics and Gynecology, James H. Quillen College of Medicine, East Tennessee State University, P.O. Box 70569, Johnson City, TN 37614, USA

## Abstract

Epidermolysis bullosa (EB) is a group of inherited blistering skin diseases that vary widely in their pathogenesis and severity. There are three main categories of EB: simplex, junctional, and dystrophic. This classification is based on the level of tissue separation within the basement membrane zone and this is attributed to abnormalities of individual or several anchoring proteins that form the interlocking network spanning from the epidermis to the dermis underneath. Dystrophic EB results from mutations in COL7A1 gene coding for type VII collagen leading to blister formation within the dermis. Diagnosis ultimately depends on the patient's specific genetic mutation, but initial diagnosis can be made from careful examination and history taking. We present a pregnant patient known to have autosomal dominant dystrophic EB and discuss the obstetrical and neonatal outcome. The paper also reviews the current English literature on this rare skin disorder.

## 1. Background

Epidermolysis bullosa (EB) is a group of inherited blistering skin diseases that vary widely in their pathogenesis and severity [1]. EB is divided into three main categories (simplex, junctional, and dystrophic) based on the level of tissue separation within the basement membrane zone [1, 2] and this is attributed to abnormalities of individual or several anchoring proteins that form the interlocking network spanning from the epidermis to the dermis underneath [3] (Table 1). EB simplex is caused by mutations in keratin 5, keratin 14, or plectin genes which result in blister formation within the basal keratinocytes [2]. The junctional forms of EB are all inherited in a recessive pattern and involve mutations in laminin 332, BPAG2, *α*6 integrin, or *β*4 integrin genes and result in skin separation at the lamina lucida [2]. Dystrophic EB results from mutations in COL7A1 gene coding for type VII collagen leading to blister formation within the dermis [2, 3]. Diagnosis ultimately depends on the patient's specific genetic mutation, but initial diagnosis can be made from careful examination and history taking [2]. We present a pregnant patient known to have autosomal dominant dystrophic EB (ADDEB) and discuss the obstetrical and neonatal outcome followed by a review of the current literature on this rare entity.

## 2. Case Illustration

**Figure 1:**
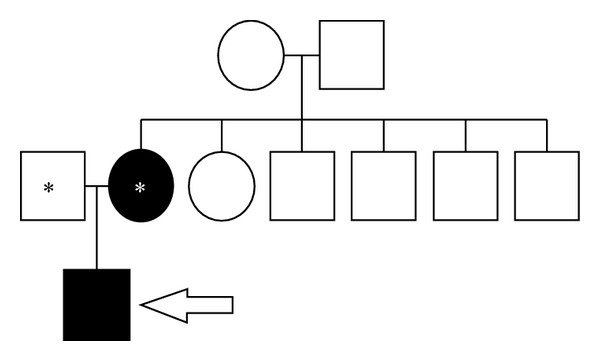
Pedigree showing the affected mother (white asterisk), unaffected partner (black asterisk), and affected infant (white arrow).

**Figure 2:**
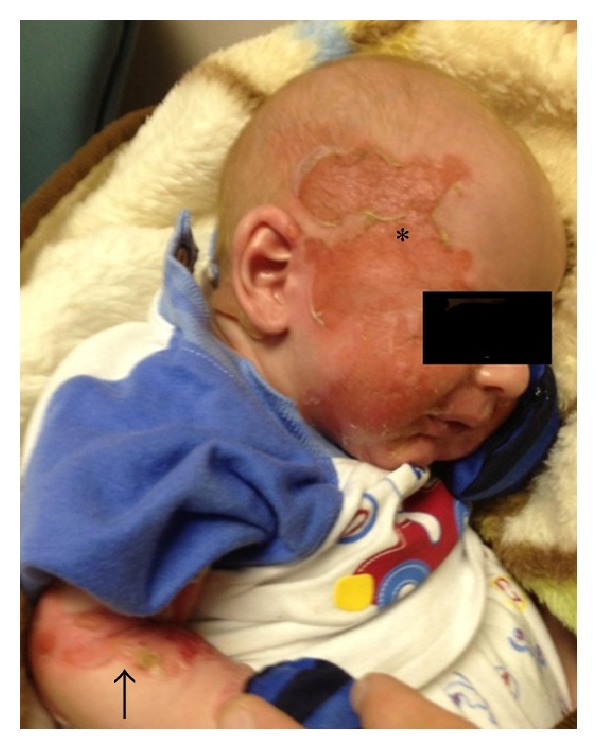
The newborn with widespread facial (asterisk) and forearm skin blistering (arrow).

**Figure 3:**
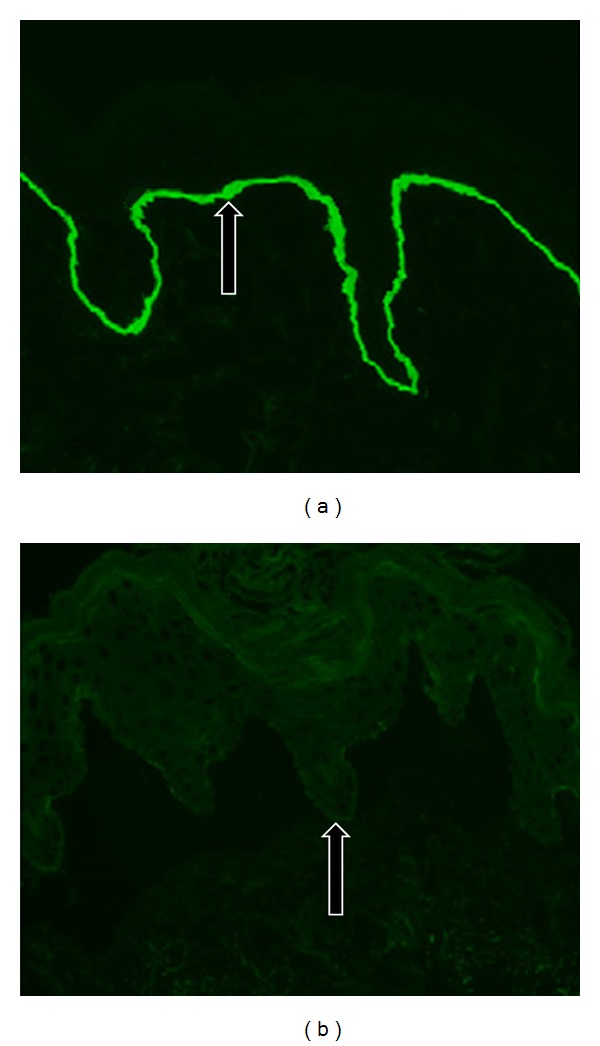
Immunofluorescence staining of the dermal-epidermal junction with antibodies to type VII collagen showing a control normal expression (a) and the minimal expression (b) of type VII collagen.

Our patient is a 19-year-old Caucasian female, G5P0040, who had routine prenatal care at our clinic. Her pregnancy had been complicated by a history of a seizure and bipolar disorders and personal history of ADDEB with documented COL7A1 gene mutation. The patient reported a family history of the disease in her maternal great aunt who was deceased; however, accurate information pertaining to the manifestations of her skin disorder could not be confirmed. The patient herself was born through a vaginal route and had been told that she had blistering and bullae formation that covered the vast majority of her body surface shortly after she was born. Throughout her early childhood years, she had recurrent skin blistering, particularly in her hands and fingers. By the age of three, she underwent surgical release of interdigit webbing between the fingers of both hands. Her disease activity diminished with advancing age thereafter. Her gingiva, buccal mucosa, teeth, and hands were normal at the time of her current pregnancy. Occasional blistering occurred with mechanical stress; however, perineal and vaginal epithelium sustained no blistering with normal vaginal intercourse. She was otherwise healthy. Her partner had no known family or personal history of EB (Figure 1—Pedigree). Throughout her prenatal care, the patient adamantly expressed her desire to undergo an elective cesarean delivery. Her desire stemmed from the fear that her infant would suffer from generalized body skin blistering if delivered vaginally as she had when she was born. She had an uneventful prenatal course. Despite having no absolute indications for a cesarean route of delivery and after proper counseling, she received intrathecal spinal anesthesia and underwent an elective low transverse cesarean delivery at 39 weeks of gestation. She delivered a male infant weighing 3000 grams with Apgar scores of 8 and 9. The immediate postoperative period was unremarkable for both neonate and mother. The patient sustained no blisters on or around her skin incision throughout her hospital stay and remained to be blister-free on her 2-week postoperative check-up. Her infant however exhibited signs of skin erythema and blistering over its back (0.5 cm), inguinal (1 cm), and right buttock areas (0.5 cm) by day 1 of life. Within 3 days, diffuse erythematous and bullous lesions progressively appeared and covered the neonate's chin, forearms, face (Figure 2), thumbs, fingers, left buttocks, remaining area of the back, and both legs covering approximately 30% of its total body surface. There was evidence of blistering and ruptured bullae but no secondary bacterial infections. Guidelines for skin care of neonates with suspected EB were implemented. These include using bland emollients, avoiding restrictive clothing and overheating, minimizing skin trauma, and rupturing of blisters using sterile needles, with topical antibiotics when needed. The differential diagnosis in this particular case was that of EB simplex versus ADDEB. A skin biopsy from the infant's back was performed and immunofluorescence mapping confirmed the suspected ADDEB in this neonate with low expression of type VII collagen in the basement membrane (Figure 3). Both patient and neonate were discharged on hospital day 4 in a stable condition. She elected to bottle feed to avoid possible nipple blistering. As of this writing, maternal health has been stable and unchanged. Except for occasional blister and bullae formation, the neonate had what seems to be a mild phenotype of ADDEB with progressive resolution of the skin lesions over the course of 4 weeks after birth. By the third month of life, the newborn had been attaining all developmental milestones and had minimal blisters and bullae with normal fingers and nail beds.

## 3. Dystrophic Epidermolysis Bullosa

Dystrophic epidermolysis bullosa (DEB) is divided into three autosomal subtypes: autosomal dominant DEB (ADDEB), recessive DEB (RDEB), and severe generalized recessive DEB (SGRDEB), previously Hallopeau-Siemens subtype (Table 1). Clinical presentation of DEB in neonates is somewhat variable with more severe blistering generally occurring in the recessive rather than the dominant subtypes.

The most common form of DEB is the autosomal dominant subtype, which is also the mildest form. Patients with ADDEB may present along a clinical spectrum ranging from localized blistering at sites of frequent trauma to generalized blisters that resolve with atrophic scarring. Generalized blistering may be seen at birth with alopecia, atrophic scarring, and dystrophic nails developing later. The majority of ADDEB cases do not present until early childhood and the course of the disease is relatively mild. One of the localized forms of ADDEB, the pretibial type, typically presents in early childhood with blistering on the pretibial skin and dorsum of the foot. Nail beds tend to be abnormal, but extracutaneous involvement is absent. Generalized forms of ADDEB, previously known as Pasini and Cockayne-Touraine variants, present at birth and are associated with milia and severely deformed or absent nail beds. Extracutaneous symptoms are reported in a minority of patients [3]. Mortality secondary to ADDEB has not been reported.

RDEB presents with blistering and erosions at birth with positive Nikolsky's sign (extension of blistering or sloughing of epidermis with application of gentle pressure to lesion or perilesional skin) and is associated with scarring, milia, and dystrophic nail beds. Atrophic scarring with alopecia, anemia, and growth retardation are common. Later in life, blistering occurs almost exclusively in folds of skin and patients frequently have mucosal manifestations of disease such as intraoral blistering and scarring that results in microstomia and ankyloglossia. Involvement of the esophagus can also occur. RDEB centripetalis is a subtype that also presents with blistering in infancy, but the blisters progress in a centripetal pattern and no intraoral or extracutaneous lesions are seen [3]. Patients with SGRDEB present with widespread blistering in infancy that involves the oral cavity and esophagus. Extensively painful and pruritic blistering results in contractures and pseudosyndactyly resulting in severe deformities. Extracutaneous manifestations are common with oral, ocular, anal, gastrointestinal, and genital mucous membrane involvement and can result in severe erosions and scarring resulting in microstomia, ankyloglossia, esophageal strictures, malnutrition, and constipation [3, 4]. Abnormal enamel and cementum lead to lifelong dental complications. The cumulative risk of death in children with RDEB is 8% by the age of 15 [5]. Sepsis, failure to thrive, and respiratory failure are the major causes of death [5]. All patients with DEB are at an increased risk of developing squamous cell carcinoma with a cumulative risk of 90% by the age of 55 [3, 4, 6].

## 4. DEB in Pregnancy

Management of pregnancy and delivery in patients with DEB can be challenging due to the limited literature available and lack of established guidelines for best practice [7]. A multidisciplinary approach involving obstetrics, anesthesiology, and dermatology is essential [6]. Many women are apprehensive about vaginal delivery because of the possibility of genital blistering and scarring and neonatal blistering, but caesarean section is not a straight-forward alternative [6, 7]. Blistering over the spine may interfere with spinal anesthesia, and trauma from intubation may result in life-threatening upper airway complications and serious postoperative complications [6–8]. The current pregnancy-related DEB literature is limited and primarily focuses on the recessive forms of DEB since those patients tend to be more severely affected and more likely to suffer from complications than individuals with ADDEB [6–10].

A literature review of DEB in pregnancy yielded several case reports and case series. The majority of reported patients have undergone vaginal deliveries, which some authors believe should be the first choice [10] despite a theoretical risk of vaginal mucosal blistering [6]. Of the five reports on pregnancy and delivery in patients with recessive DEB, eight out of ten women had vaginal deliveries without genital ulceration, vaginal scarring, or stenosis [6–10]. One of the eight women had excessive bleeding during her first pregnancy, but the remaining were uncomplicated [6–10]. Five of these women had more than one vaginal delivery, and several were able to breastfeed without nipple blistering or ulceration [6, 7]. One of the patients had a caesarean section performed due to genital ulceration, fetal growth restriction, and premature rupture of membranes with the onset of preterm labor at 36 weeks of gestation. The caesarean section was associated with blistering around the incision site, but no infection or wound separation was noted [8]. Another patient elected to have a caesarean section due to a strong fear of vaginal delivery, and she suffered no complications from the surgery [6]. A possible correlation between DEB and intrauterine growth restriction has been suggested as well [11]. In each of these reported pregnancies, a healthy neonate was delivered without evidence of skin disease [6–10]. In all deliveries, whether vaginal or caesarean delivery is attempted, special care is taken to prevent any unnecessary trauma to the skin [6].

## 5. Discussion

Our patient had a known history of ADDEB with documented COL7A1 gene mutation and delivered a neonate with suspected ADDEB that was proven on pathological evaluation. The patient's family history was only significant for a great maternal aunt with possible epidermolysis bullosa but no definitive information could be obtained to discern the subtype. In Figure 1, the patient was the only individual with the ADDEB phenotype and this was passed on to her infant. Genetic testing for COL7A1 mutation was negative in the patient's partner (biological father of the affected neonate). This suggests that the patient may have acquired a de novo mutation in COL7A1 as she tested positive for the mutation; however, the exact type of mutation was not identified. A multidisciplinary approach that involved anesthesiology, pediatrics, obstetrics, and dermatology was used in the management of this particular pregnancy. Previously published guidelines in the management of DEB in pregnancy were utilized to improve maternal and neonatal outcomes in our patient [4, 6, 12]. Given the fact that our patient had extensive bullae and blistering of her skin when she was born vaginally and the fact that her infant sustained similar lesions after being delivered by cesarean section, it seems prudent that pregnant individuals with known ADDEB may deliver vaginally and/or via cesarean section with the thought that the neonate's skin is very likely to blister. We are unable to verify which route of delivery is associated with less blistering in ADDEB as our information is limited by the retrospective nature of the mother's symptoms upon birth and the inability to verify the percent body area involved when she was born vaginally. Nonetheless for counseling purposes individuals with ADDEB should be informed about the inevitable risk of a neonate's risk of blister and bullae formation with either route of delivery. Unless absolutely necessary, we recommend that operative vaginal delivery be avoided in such situations in order to minimize mechanical trauma to the fetal head prior to delivery. In light of the available literature [6–10] and as in our patient, the vaginal mucosa of patients with EB seems to withstand regular sexual intercourse and full term labor and delivery without consequential ulceration or exacerbation of underlying disease. Cesarean section can be safely performed in patients with EB [6, 8] and may be considered for obstetrical indications [7], but neonatal skin blistering is likely to occur particularly in the ADDEB subtype as the mutation confers a definite risk on the neonate. In light of the potential complications of surgery, our patient case, the low number of reported cases of ADDEB in pregnancy, and the favorable neonatal outcomes, we do believe that a vaginal route for delivery is the safest option for both mother and infant in ADDEB.

## 6. Conclusions

ADDEB in pregnancy is a rare entity. Guidelines for its management in pregnancy are scarce at best as most of the DEB literature revolves around its recessive forms. The medical literature however provides us with a gamut of strategies to minimize blister and bullae formation in both pregnant individuals and neonates alike with DEB. In this case report, we reflect upon the challenges that ADDEB poses on any pregnancy. Whether a vaginal or cesarean route is chosen, neonates of mothers with COL7A1 mutation and ADDEB seem to be at a definite risk of sustaining skin blisters and bullae after delivery. Given no obstetrical indications for a cesarean delivery, the vaginal route for delivery is most likely the safest option. Accordingly, proper counseling of the risks and benefits to maternal and neonatal health is the key in individualizing management plans in pregnancies complicated by ADDEB.

## Figures and Tables

**Table 1:** Classification of common inherited epidermolysis bullosa (EB) phenotypes.

Major type	Inheritance	Major subtype	Protein/gene involved	Level of cleavage	Manifestations
		*EBS Weber-Cocayne *			(i) Onset in late childhood or early adulthood (ii) Blisters on palms and soles
EB simplex (EBS)	Autosomal dominant	*EBS Koebner *	K5, K14	Intraepidermal	(i) Present at birth (ii) Worse blistering on extremities
		*EBS Dowling-Meara *			(i) Onset in infancy (ii) Herpetiform grouping of blisters (iii) Symptoms improving with age (iv) Infant mortality rate of 2.8% in first year of life
		*EBS muscular dystrophy *	Plectin		(i) Onset in late childhood (ii) Generalized blistering and muscle weakness

		*JEB Herlitz *	Laminin 5	Intralamina densa	(i) Present at birth (ii) Intractable blistering and erosions (iii) High infant mortality (40% in first year of life and rising to 61.8% by the age of 15)
Junctional EB (JEB)	Autosomal recessive	*JEB non-Herlitz *	Laminin 5, type XVII collagen		(i) Present at birth (ii) Milder form of JEB with minimal extracutaneous involvement (iii) High infant mortality (40% in first year of life and rising to 61.8% by the age of 15)
		*JEB pyloric atresia *	*Α*6*β*4 integrin	Intraepidermal and/or intralamina densa	(i) Pyloric atresia (ii) Wide variety in severity of blistering (iii) Often fatal at early age

	Autosomal dominant	*Dominant DEB *			(i) Onset at birth or in early childhood (ii) Uncommon extracutaneous manifestations (iii) Abnormal nails and atrophic scarring
Dystrophic EB (DEB)	Autosomal recessive	*Recessive DEB Hallopeau-Siemens (HS) *	Type VII collagen	Sublamina densa	(i) Present at birth (ii) Extensive scarring (iii) Extracutaneous involvement (iv) Increasing risk of SCC (v) 8% mortality risk by the age of 15
		*Recessive DEB non-Hallopeau-Siemens *			(i) Present at birth (ii) Scarring being less severe than in HS type (iii) Extracutaneous manifestations that may be present

## References

[1] Pfendner EG, Nakanol A, Pulkkinen L, Christiano AM, Uitto J (2003). Prenatal diagnosis for epidermolysis bullosa: a study of 144 consecutive pregnancies at risk. *Prenatal Diagnosis*.

[2] Sawamura D, Nakano H, Matsuzaki Y (2010). Overview of epidermolysis bullosa. *Journal of Dermatology*.

[3] Das BB, Sahoo S (2004). Dystrophic epidermolysis bullosa. *Journal of Perinatology*.

[4] Bruckner-Tuderman L (2010). Dystrophic epidermolysis bullosa: pathogenesis and clinical features. *Dermatologic Clinics*.

[5] Fine J, Johnson LB, Weiner M, Suchindran C (2008). Cause-specific risks of childhood death in inherited epidermolysis bullosa. *Journal of Pediatrics*.

[6] Baloch MS, Fitzwilliams B, Mellerio J, Lakasing L, Bewley S, O’Sullivan G (2008). Anaesthetic management of two different modes of delivery in patients with dystrophic epidermolysis bullosa. *International Journal of Obstetric Anesthesia*.

[7] Hanafusa T, Tamai K, Umegaki N (2012). The course of pregnancy and childbirth in three mothers with recessive dystrophic epidermolysis bullosa. *Clinical and Experimental Dermatology*.

[8] Bianca S, Reale A, Ettore G (2003). Pregnancy and cesarean delivery in a patient with dystrophic epidermolysis bullosa. *European Journal of Obstetrics Gynecology and Reproductive Biology*.

[9] Choi SD, Kho YC, Rhodes LM, Davis GK, Chapman MG, Murrell DF (2011). Outcomes of 11 pregnancies in three patients with recessive forms of epidermolysis bullosa. *British Journal of Dermatology*.

[10] Büscher U, Wessel J, Anton-Lamprecht I, Dudenhausen JW (1997). Pregnancy and delivery in a patient with mutilating dystrophic epidermolysis bullosa (Hallopeau-Siemens type). *Obstetrics and Gynecology*.

[11] Ozkaya E, Baser E, Akgul G, Kucukozkan T (2012). A pregnancy complicated with fetal growth restriction in a patient with dystrophic epidermolysis bullosa. *Journal of Obstetrics and Gynaecology*.

[12] Gonzales M (2013). Evaluation and treatment of the newborn with epidermolysis bullosa. *Seminars in Perinatology*.

